# *Blepharostoma trichophyllum* S.L. (Marchantiophyta): The Complex of Sibling Species and Hybrids

**DOI:** 10.3390/plants9111423

**Published:** 2020-10-23

**Authors:** Vadim A. Bakalin, Anna A. Vilnet, Seung Se Choi, Van Sinh Nguyen

**Affiliations:** 1 Laboratory of Cryptogamic Biota, Botanical Garden-Institute FEB RAS, Vladivostok 690024, Russia; vabakalin@gmail.com; 2 Laboratory of Flora and Vegetation, Polar-Alpine Botanical Garden-Institute Kola SC RAS, Apatity 184209, Russia; anya_v@list.ru; 3 Team of National Ecosystem Survey, National Institute of Ecology, Seocheon 33657, Korea; 4 Institute of Ecology and Biological Resources, Graduate University of Science and Technology, Vietnam Academy of Science and Technology, Ha Noi 10000, Vietnam; vansinh.nguyen@iebr.ac.vn

**Keywords:** *Blepharostoma*, East Asia, Pacific Asia, stasis, molecular phylogenetics, ITS1-2 nrDNA, trn*L*–*F* cpDNA

## Abstract

*Blepharostoma trichophyllum* was found to be a *species collectiva* formed by several strongly genetically different species. The taxonomic diversity in the group is the possible result of radiation in early stages; then, these taxa likely survived for a long time in similar environmental conditions, which resulted in stasis. Presently, the existing taxa are similar one to another and may be morphologically distinguished with difficulties. The most taxonomically valuable morphological characteristics include oil bodies and cells in the leaf segment features. The most diverse genotypes (the vast majority of which are treated here as distinct species) were found in amphi-Pacific Asia, which may reflect the evolutionary history of the genus or may be the consequence of more profound sampling in the macro-region in comparison with other parts of the Holarctic.

## 1. Introduction

*Blepharostoma* (Dumort.) Dumort. is a non-speciose genus that occurs frequently in various types of humid and mesic vegetation communities. It is largely restricted to the Holarctic [1,2,3,4], where it spreads to high arctic extremes. Southwards, the genus reaches the Paleo- and Neotropics in Africa [5], Mexico and South America [1], and Indochina (the present paper). The genus representatives occur mostly on organic substrates with a slow process of decomposing, such as humus, decaying wood, and dead bryophyte patches. More rarely, *Blepharostoma* is observed on moist rocky substrates. The genus currently includes four species [6], of which one only—*B. trichophyllum* (L.) Dumort.—has undoubted status, whereas the others (*B. arachnoideum* M.Howe, *B. indicum* G.Asthana, M.Saxena et Maurya, and *B. minor* Horik.) have a ‘knowledge problem’ status (two asterisks in l.c.). *B. trichophyllym* admittedly possesses a wide Holarctic range, whereas other taxa are locally distributed.

*Blepharostoma* appears to be the one of the oldest genera of leafy liverworts, as shown by Laenen et al. [7]. The estimated age of this genus is approximately 160 Ma. A similar age was suggested for species including the simple thallose *Metzgeria* Raddi (167 Ma), the oligotypic *Phyllothallia* E.A.Hodgs. (158 Ma), and the complex thallose monospecific genus *Neohodgsonia* Perss. (151 Ma). The basal position of the genus *Blepharostoma* in the suborder Lophocoleineae was repeatedly proven in several molecular phylogenetic studies based on different combinations of DNA loci from a single sampled specimen of *B. trichophyllum* originating from Finland, Slovenia, and the USA [7,8,9]. The hidden taxonomic diversity was alluded to by Hassel et al. [10] during their test of molecular markers for plant barcoding, including five *Blepharostoma trichophyllum* specimens composed of two clades treated as *B. trichophyllum* and *B. trichophyllum* subsp. *brevirete* (Bryhn et Kaal.) R.M.Schust., as well as the phylum, which was provisionally assigned to a new taxon (not described in l.c.).

The generic features of *Blepharostoma* include (1) plants small and delicate, (2) nearly isophyllous, (3) leaves transversely inserted, (4) those divided into uniseriate cilia-like lobes called segments, (5) gemmae from the apices of the leaf and underleaf segments, and (6) somewhat reduced seta (8 epidermal and 4 inner rows of cells) [1]. Due to this combination of features, the genus by itself is easy to recognize, and the main problem is in the delimitation of infra-generic entities.

The delimitation problem was expected by us when we sought to identify the distinct taxonomic position of morphologically intermediate ‘phases’ between specimens related to *B. trichophyllum* subsp. *trichophyllum* and its subsp. *brevirete*. Surprisingly, the genetic diversity was much higher than assumed. Considering this genetic difference, we undertook wider sampling in the ‘*B. trichophyllum* s.l.’ complex based mostly on specimens gathered in Pacific Asia. As a result, we found great morphological variation in the comparative lengths of the cells in the leaf segments, protrusions of the cross walls of the segment cells, sizes and numbers of the oil bodies, and possible stem cross-section features. All of those variations cannot be ascribed only to the variability of *B. trichophyllum*. This raised the question as to whether this variation passes species limits or whether there are several species-level taxa within this complex. We selected specimens from distant regions of East Asia and processed them via integrative research, including molecular–genetic, morphology, and geographical analyses. Describing the newly revealed taxa of this complex was the main goal of the present study.

## 2. Material and Methods

### 2.1. Specimen Collection

In total, 76 specimens of the *Blepharostoma trichophyllum* complex were analyzed (Table 1). Most of them were from the Russian Far East and adjacent countries, such as China, Japan, South Korea, and Vietnam, and a few that were from Siberia and the European part of Russia, Norway, and the USA. Five specimens of *B. arachnoideum* were also involved in the examination. Additionally, six accessions of *Blepharostoma trichophyllum* from Finland, Norway, and Greenland and 21 accessions of the outgroup taxa were taken from GenBank. In total, 87 specimens of *Blepharostoma* and 16 specimens of the outgroup taxa comprise the dataset. The outgroup species are represented by 8 from 12 known families of the suborder Lophocoleineae, according to Söderström et al. [6]. Trees were rooted on *Hygrobiella nishimurae* N. Kitag. from the phylogenetic allied suborder Jungermanniineae. The composition of the outgroups in the ITS1-2 and *trn*L-F datasets depended on presence of appropriate sequence data in GenBank. The voucher details, preliminary sample identification from morphological evidence, and the GenBank accession numbers are presented in Table 1 and Table 2.

When performing the molecular genetic analysis, even first stages revealed the strong genetic diversity within the genus, so we were compelled to find features that might be useful to compare the morphology with the molecular-genetic analysis results. The intuitive selection of features in sterile gametophytes (since the generative structures and sporophytes were rarely available) resulted in a list of the following features used to compile the primary matrix:

(1) oil bodies size, shape, and number per cell;
(2) the actual size and relative proportions of the cell length/width ratio in the middle part of the leaf segment. This parameter was measured as the size variation between the three middle cells in the cilia (thus, neither basal nor apical cells were taken into account);
(3) middle cells in the leaf segments’ cross wall protrusions (dilation). These cells were called dilated if inter-cellular interval (cross wall junction in Wagner [11]) was wider than the cell width and made the cilia’s surface somewhat crenulate (not straight);
(4) the stem’s cross-section features, including the cross-section’s actual size, the relative height of the section (measured as the cell number), the trigones, and the wall thickness distribution.

A vast majority of the sequenced specimens were studied when they were alive, and photographs of living cells were obtained, along with photographs of the *Blepharostoma* patches under living conditions. These photographs were supplemented with photographs of the morphology of the plants based on herbarium specimens and together formed illustrative confirmations of the discussed taxa.

### 2.2. DNA Isolation, Amplification and Sequencing

A NucleoSpin Plant Kit (Macherey-Nagel, Düren, Germany) was used to extract DNA from the dried liverwort tissue. The primers suggested by White et al. [12] for ITS1-2 and Taberlet et al. [13] for *trn*L-F were implemented for amplification and sequencing.

PCR was carried out in 20 µl volumes with the following procedure: 3 min at 94 °C, 30 cycles (30 s 94 °C, 40 s 56 °C, and 60 s 72 °C), and 2 min for the final extension time at 72 °C. Amplified fragments were visualized on 1% agarose TAE gels by EthBr staining and then purified with GFX PCR DNA and Gel Band Purification Kits (Amersham Biosciences, Chicago, IL, USA). The sequencing reactions were performed with the ABI Prism BigDye Terminator Cycle Sequencing Ready Reaction Kit (Applied Biosystems, Waltham, MA, USA) following the standard protocol provided for the 3730 DNA Analyzer (Applied Biosystems, Waltham, MA, USA) at the Genome Center of EIMB (Moscow, Russia).

### 2.3. Phylogenetic Analysis

The newly obtained sequences were assembled in BioEdit 7.0.1 [14], and then ITS1-2 and *trn*L-F nucleotide datasets were automatically aligned using full multiple alignment with default settings for the gaps and extension weights via the ClustalW tool with subsequent manual corrections. Topological incongruence was revealed among the datasets during the preliminary phylogenetic estimations. Thus, the analyses were implemented for each dataset separately. All positions of the final alignments were included in the calculations, and absent data at the ends of the regions were coded as missing.

Phylogenies were tested by the maximum parsimony (MP) method with TNT v. 1.5 [15] (Goloboff and Catalano, 2016), maximum likelihood (ML) with PhyML v. 3.0 [16], and Bayesian reconstruction with MrBayes v. 3.2.1 [17]. The parsimony analysis with TNT involved a New Technology Search for the minimal-length tree using five iterations and 1000 bootstrap replicates. Default settings were used for the other parameters, and gaps were treated as missing.

For the ML analysis, ModelGenerator [18] identified GTR+I+Г as the best-fitting evolutionary model for each dataset. Gamma distribution with four rate categories was used to handle the among-site rate heterogeneity. Bootstrap support (BS) for individual nodes was assessed using a resampling procedure with 500 replicates. According to the stopping frequency criterion (FC) for the bootstrap [19], the ITS1-2 dataset should require 350 replicates to reach convergence with a Pearson average ρ100 = 0.992385 and *trn*L-F and 400 replicates with ρ100 = 0.992489, as estimated by RAxML v. 7.2.6 [20].

The Bayesian estimation for each dataset was done with the GTR+I+Г model, and gamma distributions were approximated using four rate categories. Two independent runs of the Metropolis-coupled ΜCMC were used to sample the parameter values in proportion to their posterior probability. Each run included three heated chains and one unheated chain, and two starting trees were chosen randomly. Chains were run for ten million generations, and trees were sampled every 1000th generation. The first 2500 trees in each run were discarded as burnin. Thereafter, 15,000 trees were sampled from both runs. For ITS1-2, the software tool Tracer [21] revealed the effective sample size (ESS) as 7050.0732 and the auto-correlation time (ACT) as 2553.4487. The average standard deviation of the split frequencies between two runs was 0.004277. For *trn*L-F, the ESS was 6427.4931, the auto-correlation time (ACT) was 2800.7809, and the average standard deviation was 0.003623. Bayesian posterior probabilities were calculated from the trees sampled after burn-in.

The average pairwise p-distances were calculated to test the level of the infrageneric and infraspecific variability of ITS1-2 and *trn*L-F in the genus *Blepharostoma* using the pairwise deletion option for counting gaps in Mega 5.1 [22].

## 3. Results

In total, ITS1-2 nucleotide sequences were newly obtained for 71 specimens, *trn*L-F sequences were obtained for 77 specimens, and all were deposited into GenBank. The ITS1-2 dataset of *Blepharostoma* and the outgroup taxa consisted of 84 specimens and 937 sites, while *trn*L-F consisted of 90 specimens and 588 sites. The number of constant positions in ITS1-2 and *trn*L-F was 316 (33.72%) and 250 (42.51%), respectively, the number of variable positions was 601 (64.14%) and 316 (53.74%), and the number of parsimony-informative positions was 447 (47.74%) and 251 (42.69%).

For the ITS1-2 dataset, the MP analysis yielded 33 equally parsimonious trees with a length of 2157 steps (CI = 0.640808 and RI = 0.864219). The ML estimation produced a tree with a Log likelihood of −7988.1921. The arithmetic means of the Log likelihoods in Bayesian analysis for each sampling run were −8060.69 and −8055.55. For the *trn*L-F dataset in the MP analysis, 36 equally parsimonious trees with a length 836 steps were obtained (CI = 0.616390 and RI = 0.862845). The tree with a Log likelihood of −4822.0877 was calculated in ML, and, in the Bayesian analysis, the means of the Log likelihood for each sampling run were −4845.80 and −4850.43.

**Figure 1:**
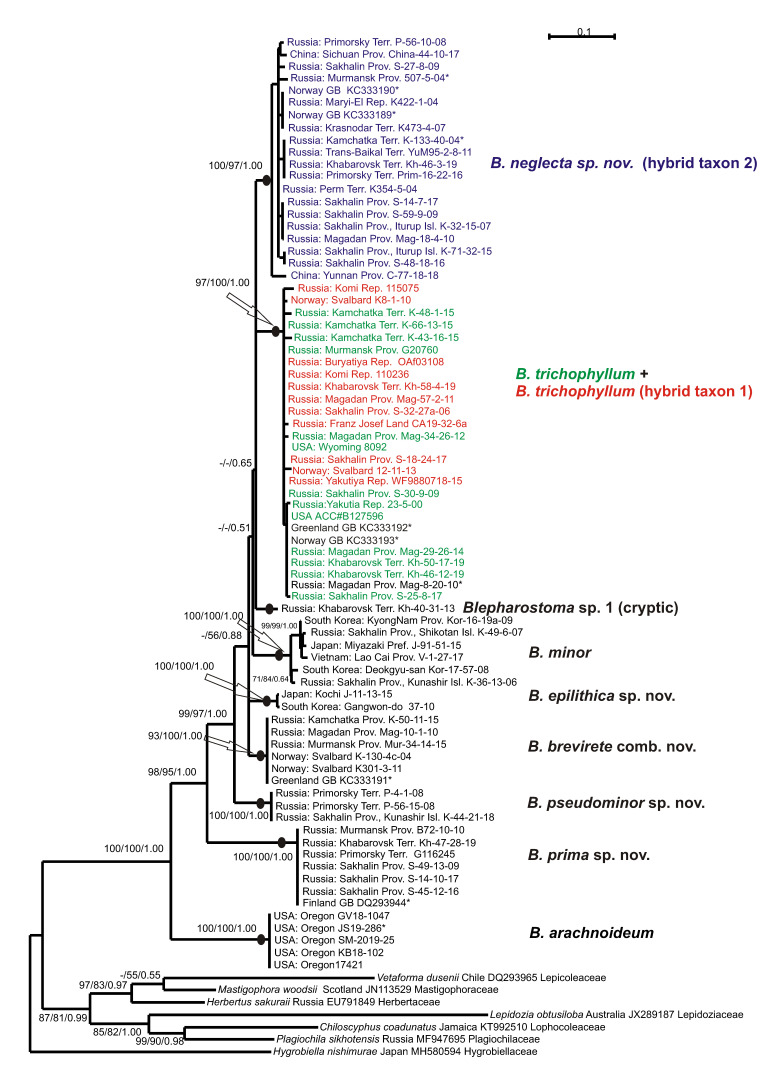
Phylogram obtained under the maximum likelihood criterion for the genus *Blepharostoma* based on the ITS1-2 nrDNA. Bootstrap support values are indicated under maximum parsimony, maximum likelihood, and Bayesian posterior probabilities of >50% (0.50).

**Figure 2:**
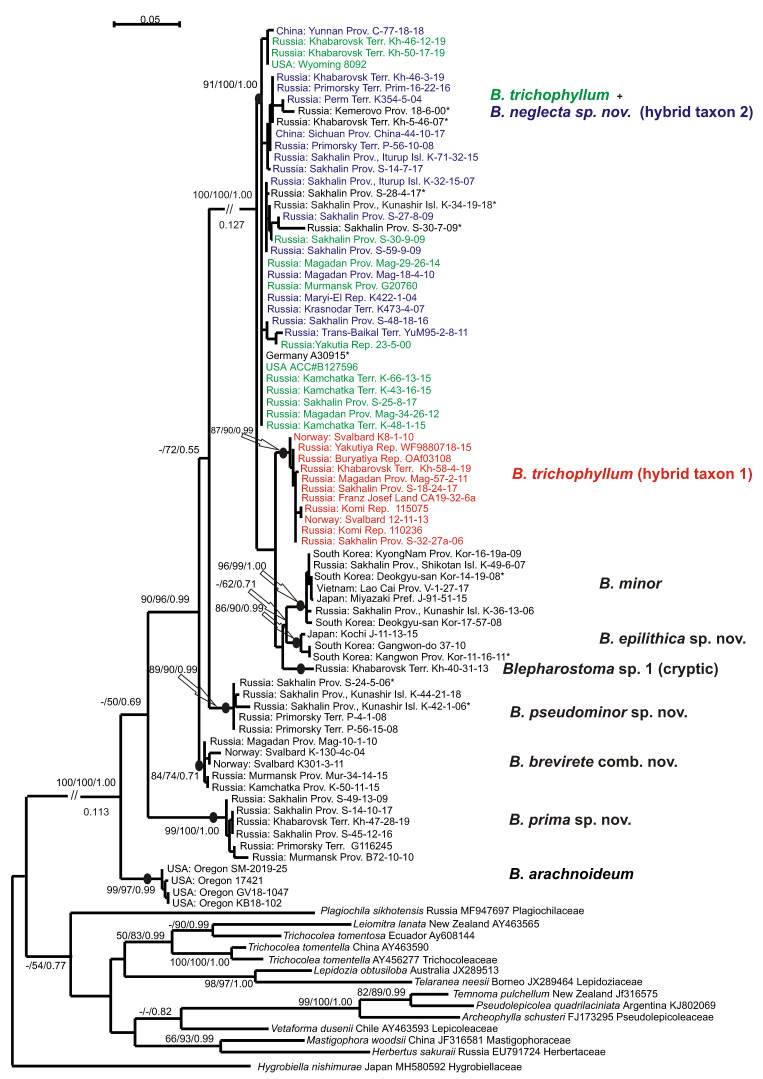
Phylogram obtained under the maximum likelihood criterion for the genus *Blepharostoma* based on the *trn*L-F cpDNA. Bootstrap support values are indicated under maximum parsimony, maximum likelihood, and Bayesian posterior probabilities of >50% (0.50). The lengths of the cut branches are shown.

The tree topologies for each dataset revealed under the three criteria appear highly congruent. Figure 1 shows the single tree for the ITS1-2 dataset retained under ML, along with the ML and MP bootstrap values (BS) and the Bayesian posterior probabilities (PP) for each node. Figure 2 shows the tree for the *trn*L-F dataset.

Eight main clades and phyla could be distinguished on each tree topology, but the affinity of the clades was differing and had poor support. The basal position in the *Blepharostoma* phylogeny for both topologies belonged to North American *B. arachnoideuim* (BS = 100% in MP, BS = 100% in ML, PP = 1.00 in BA, and 100/100/1.00 in the ITS1-2 and *trn*L-F trees) in four/five specimens that composed a strongly supported clade (100/100/1.00 in ITS1-2, 99/97/0.99 in *trn*L-F). The next divergence (98/95/1.00 in ITS1-2 -/50/0.69 in *trn*L-F) belonged to the clade with six/seven specimens (100/100/1.00 in ITS1-2, 99/100/1.00 in *trn*L-F) from Murmansk, Sakhalin Provinces, Primorsky, Khabarovsk Territories, and in a specimen from Finland (DQ293944), tested in the liverwort phylogeny by He-Nygren et al. [9] under *B. trichophyllum*. In the current study, these specimens relate to a new species, *B. prima* sp. nov. The following relations in both topologies are different and obtained partial support, so the evolution within each genus could not be clarified directly. The next divergence in the ITS1-2 tree (99/97/1.00 belonged to the clade with three specimens from Primorsky Territory and Kunashir Island (100/100/1.00), classified here as *B. pseudominor* sp. nov. The divergence of this clade by *trn*L-F occurred later and was poorly supported (−/72/0.55). This clade united five specimens from the same regions (89/90/0.99), two of them with only *trn*L-F sequence data. The follow divergence on ITS1-2 (-/56/0.88) showed a clade (100/100/1.00) with six specimens from Svalbard, Murmansk, and Magadan Provinces, Kamchatka, and a specimen from Greenland, provisionally assigned to a new species by Hassel et al. [10] (2013). Under the *trn*L-F topology, this clade (84/74/0.71) diverged earlier (90/96/0.99). Based on the morphological evidence, all specimens of this clade were assigned to species *B. brevirete* comb. nov. The next three subsequently divergent clades on the ITS1-2 topology united into one unsupported clade on the *trn*L-F topology. Two/three specimens from Japan and South Korea composed a clade (100/100/1.00 in ITS1-2 and 86/90/0.99 in *trn*L-F), and their morphological and ecological features allowed us to describe a new species—*B. epilithica* sp. nov. The clade (100/100/1.0 in ITS1-2, 96/99/1.00 in *trn*L-F) composed of six/seven specimens with predominantly Asian distribution (Russian Far East, Japan, South Korea, and Vietnam) that morphologically resembled *B. minor*. The single specimen from Khabarovsk Territory with a combination of unique features in both the ITS1-2 and *trn*L-F sequences was distinguished by the cryptic taxon tentatively named *B. sp.* until further consideration. More than half of the tested *B. trichophylum* s.l. specimens from the USA, Norway, Greenland, Germany, China, and different regions of Russia take the terminal position on the tree topologies in the two colored clades. The green colored specimens on the ITS1-2 tree intermingled in one clade with the red ones (97/100/1.00), and those on *trn*L-F intermingled with the blue ones (91/100/1.00). The green colored specimens were morphologically similar to *B. trichophyllum* and became one of the parent species for “blue” and “red” taxa that hybridized with two unknown species. According to the obtained topology, the green- and red-colored specimens possess a common ITS1-2 (father inheritance), and the green and blue-colored specimens possess a common *trn*L-F (maternal inheritance). The clade on the *trn*L-F tree with red-colored specimens called the *B. trichophyllum* hybrid taxon 1 (87/90/0.99) has a similar morphology to *B. brevirete* and appears to be a cryptic taxon. The clade on the ITS1-2 tree with blue-colored specimens characterized by unique morphological features is related to the new species *B. neglecta* sp. nov. (hybrid taxon 2) (100/97/1.00). On the ITS1-2 topology, for three specimens in the “green–red” clade marked by a black color and asterisk, only ITS data were obtained. Among them, the specimens from Hassel et al. [10] originated from Greenland (KC333192) and Norway (KC333193) and were cited previously as *B. trichophyllum* subsp. *brevirete*. Two *B. trichophyllum* specimens from Norway (KC333190, KC333189) tested in this study were placed in the “blue” *B. trichophyllum* hybrid taxon 2. On the *trn*L-F tree in the “green–blue” clade, six specimens were marked by a black color and asterisk due to the presence of only cpDNA data among them. As we did not determine the “green parent”, we suggest two distinct species of “blue” and “red” that are widely distributed on common areas. The presence of single parental types of ITS1-2 in “red” hybrid accessions indicates complete evolution and could suggest ancient hybridization in the *B. trichophyllum* complex, as well as the possible extinction of unknown parents compared to the modern hybridization process between two species with sympatric distribution in the phylogenetically young genus of *Barbilophozia* Loeske [23]. To obtain additional support for the taxon delimitation and trends of evolution in the genus *Blepharostoma*, the infraspecific and infrageneric *p*-distances were calculated (Table 3). The level of infraspecific variability was mainly low in both studied loci (0–1.4% (2% only for *B. minor*) in ITS1-2, and 0.2–1.1% in *trn*L-F), whereas the *p*-distances between the obtained clades were much higher, so such clades could be treated as distinct species. *Blepharostoma arachnoideum*, the first divergent species, revealed the highest level of nucleotide sequence divergence from other taxa—18.5–21.9% in ITS1-2 and 7.7–13.1% in *trn*L-F. The second divergent species was *B. prima,* with 15.3–18.5% in ITS1-2 and 8.8–13.4% in *trn*L-F. The complex of the following species evidently diverged later and will be discussed separately from the basal species. Two other species with high infrageneric *p*-distances—*B. pseudominor* (7.7–11.2% in ITS1-2 and 3.3–9.0% in *trn*L-F) and *B. minor* (8.4–11.2% in ITS1-2 and 3.5–7.9% in *trn*L-F)—passed unclear positions on the phylogenetic trees. Indirectly, both *B. minor* and *B. pseudominor* can be characterized by rapidly evolving ITS1-2, whereas the rate of *trn*L-F’s evolution is similar to its related species. *B. brevirete* has a similar level of differentiation from other species with both loci (5.7–8.4% in ITS1-2 and 3.3–7.4% *trn*L-F). Two species *B. epilitica* and “*B. sp.*” evidently originated later then *B. minor* and *B. pseudominor* but kept the same characteristics of quickly evolving ITS1-2 (5.8–10.2% and 5.7–9.4%, respectively) and slowly evolving *trn*L-F (3.2–8.8 and 2.7–6.8%, respectively). The three taxa of the colored *B. trichophyllum* clades appear to be three distinct species, two of which are characterized by hybrid origins with unknown parents. We considered the absence of secondary parents as additional evidence for the species status of the hybrids, rejecting modern hybridization between existing species. The variability in ITS1-2 between “green” and “red” clades (1.4%) corresponds to the level of infraspecific variability in other *Blepharostoma* species between the “green+red” and “blue” clades (8.1–8.3%), for the level of infrageneric variability in *Blepharostoma*; the variability in *trn*L-F between the “green” and “blue” clades (0.9%) compared to infraspecific variability; and between the “green + blue” and “red” clades (2.6–3.2%) compared to the level of infrageneric variability in *Blepharostoma.* Below, we provide the revisions of the species descriptions, establish four novel species, new combination, and discuss the evidence of hybridization and cryptic speciation.

## 4. Discussion

### 4.1. Morphology

The morphological similarities of the revealed genotypes compelled us to treat all phylogenetic lines as subspecies or variations of the widely accepted *Blepharostoma trichophyllum*. However, the high genetic distances (Table 3) are comparable with the inter-generic differences in some groups of hepatics and are certainly higher than the commonly observed inter-specific variations. The latter variations are largely determined by the ancient origin of *Blepharostoma*. For example, *Diplophyllum* (Dumort.) Dumort. is ca. 53 Ma old [7] and contains ca. 20 species [6], uniting taxa that are different by 4.3–14.0% in ITS1-2 and by 3.0–6.5% in *trn*L-F, as estimated by sampling in Bakalin et al. [24]. *Scapania* (39 Ma, ca. 100 species) possesses inter-specific differences of 5.8–6.9% in ITS1-2 and 4.7–5.6% in *trn*L-F among the most ancient taxa [25]. *Hygrobiella* (31 Ma, 4 species) has distances of 4.5–9.0% in ITS1-2 and 2.1–3.6% in *trn*L-F [26]. Although *Blepharostoma* features a low evolution rate among its morphological features (or its morphological similarities are the result of convergent evolution), all units found on the phylogenetic trees can be robustly treated as genetically distinct species. The absence of at least two parent species and the restricted areas of existing species could reflect a reduction in previous species diversity over geological time (the same is true for the possible extinctions discussed by Laenen et al. [7]).

Within the genus, *B. arachnoideum* is only distinctly morphologically different from other congeners. The essential features distinguishing the species were discussed by Wagner [11] and include collapsed leaf segment cells in dry conditions and commonly branched leaf segments that are “seen on every shoot in robust plants, with as many as half the leaves with a forked segment in well-developed material” [11] (p. 697). These two features classify *B. arachnoideum* distinctly apart from other known congeners. Currently, *B. arachnoideum* is a locally distributed and rare taxon restricted to the Pacific coast of North America featuring boreal and cool temperate climates. The distance between *B. arachnoideum* and *B. trichophyllum* s.l. is the highest in the genus and reaches 18.5–21.9% in ITS1-2 and 7.7–13.1% in *trn*L-F, which potentially allows segregating the various subgenera for this species. However, we did not perform such a segregation to prevent superfluous complication of the taxonomic structure of the genus.

One more taxon, *B. minor*, which is recognized at the species level in the last word checklist [6], likely belongs to the *B. trichophyllum* s.l. complex, as shown in the present analysis. The remaining *B. indica* [27] is a taxon whose taxonomic position we could not identify with certain confidence. We suggest that this poorly known species may belong to the *B. trichophyllum* s.l. complex of taxa but does not belong to any of the taxa recognized in the present paper. *Blepharostoma indica* is characterized by small, smooth-surfaced oil bodies that are few in number (3–4 per cell, ca. 2 μm in the diameter), a combination that is not known in other taxa of the genus. Moreover, the plant’s 2–3-lobed leaves (if not the result of plant ‘weakness’) and verrucose (not striolate or papillose) leaf cuticle differentiate it from other taxa of *B. trichophyllum* s.l. Considering the revealed genetic diversity within *B. trichophyllum* s.l. over the course of the present study, *B. indica* may be treated as a possible Western Himalayan endemic species whose position in the taxonomic structure of the genus needs further consideration.

As seen from the results, the genetic diversity was (and, probably, is) still poorly understood for *B. trichophyllum* s.l. The phylogenetic analysis showed at least seven well-supported clades, with two of them possessing hybrids based on the analysis of ITS1-2 and *trn*L-F. The largest variation in the studied material was seen in the numbers, sizes, and surface features of oil bodies in the leaf segment cells; the relative lengths of cells in the cilia; and the ecology and distribution. The vast majority of morphological parameters freely intergrade with one another, which suggests that all revealed taxa should be considered subspecies, not species. However, the latter is not possible if the *p*-distances are considered. Moreover, to accept the subspecies status of all revealed entities, (1) *B. minor* should be synonymized with *B. trichophyllum* s.l. and (2) the information on the distribution of old taxa would be lost, as subspecies are rarely revealed in purely floristic studies. Moreover, taking into account the possible low evolution speed in the genus, each entity may have a unique and long history of dissemination that will help reconstruct the history of its geographic dissemination, not only for the genus by itself but also for some other groups of liverwort in their early stages.

### 4.2. Phytogeographic Considerations

Laenen et al. [7] estimated the approximate origin time of the genus *Blepharostoma* as far back as 160-198 Ma. The species diversification time within the genus, however, was not suggested. Judging from the obtained tree, the first result of speciation was the splitting of *B. arachnoideum,* followed by diversification within *B. trichophyllum* s.l. Since the support of the tree backbone is low while the clades corresponding to the species are highly supported, the taxa within *B. trichophyllum* s.l. may be regarded as the result of evolutionary radiation at early stages and not associated with morphological disparity. Struck et al. [28] regarded this situation as evidence of stasis, identified as (l.c., p. 155): the “retention of the same ancestral character state over an extended period”. Indeed, Figure 2 (D) in [28] shows a scheme similar to our results. This is a widely known problem in taxonomy, where genetically different units are very similar in their morphology (a remarkable example of this problem is *Blepharostoma*). Minelly [29] mentioned this problem as one of the most vital challenges in the future systematic management of nomenclature for provisionally circumscribed taxa. Moreover, as Minelly [29] (p. 14) wrote (according to [30]), “the status of cryptic species does not describe a natural phenomenon, but only a temporary problematic formalization of species delineation”. For the circumscribed situation within *Blepharostoma*, we described some taxa as new and some as cryptic, understanding that this is not the ‘fault’ of the species if we could not find robust distinguishing features between them.

Since the current distribution of *B. trichophyllum* s.l. is not yet well-known, we cannot estimate with certain confidence the reasons, times, and areas that promoted speciation in the group. However, we suggest that some of the extant taxa within *B. trichophyllum* s.l. are the result of diversification correlated to the collision of the Indian subcontinent with Asia and the splitting of ancient Asian monsoons into East Asian and Southeast Asian monsoons [31,32,33] (likely from the middle Miocene), where the East Asian monsoon collided (and continued to collide) with the humid and mild oceanic climate in the amphi-Pacific Northeast Asia and insular part of East Asia. This sudden change in climate, against a background of uneven cooling from the Miocene (thus nearly synchronous with East Asian monsoon appearance) and the effect of insularity in the current hemiarctic-cool- to warm-temperate edges of East Asia, reinforced by volcanic eruptions in the northwestern flank of the Pacific Ring of Fire, could promote speciation in the treated area as it also occurred in other liverwort groups [34,35]. The coincidence of vegetation zones and even longitudinal segments with the distribution range of some of the revealed taxa may be regarded as a possible confirmation of this hypothesis.

Although the distribution of all recognized taxa is poorly understood, some preliminary observations can be made, taking into account the distribution data obtained in Pacific Asia. In general, the observed regularity is similar to that found in the distribution patterns of three species of *Hygrobiella*, as we described before [36], including two main features: (1) a distribution of morphologically similar taxa that is partly sympatric and partly allopatric and (2) genetic and taxonomical diversity that is concentrated in the wide contact zone between hemiboreal, boreal, and hemiarctic zones in the amphi-Pacific areas around the Sea of Okhotsk. Conversely, this may be the simple consequence of deeper sampling from that area. *Blepharostoma trichophyllum* s.l. has a less definite diversity center, and, unlike *Hygrobiella*, we cannot determine with confidence whether we found the taxonomic diversity center of the genus or simply molecular diversity in the area, which is only one of many areas with high genetic diversity of this genus in the Holarctic.

The most common taxon in the area, recorded from Magadan Province to Southwest China is *Blepharostoma neglecta* (hybrid 2). Its mother’s taxon (*B. trichophyllum*) is distributed in East Asia more narrowly than its descendant and has been hitherto recorded only around the Sea of Okhotsk (although it is widely distributed in Europe and North America). The *B. trichophyllum* hybrid 1 is characterized by a similar distribution to its father taxon (*B. trichophyllum*). *Blepharostoma minor* is widely distributed on rocky substrates from hemiboreal (in South Kurils) to subtropical zones located southward but restricted to higher elevation belts. This species was not confirmed genetically in southern China, where it should be distributed.

The southern Japanese–southern Korean distribution is characterized by *B. epilithica* found on cliffs in warm–temperate communities. *Blepharostoma brevirete* was confirmed only in Northeast Asia (in Pacific Asia). In general, this species is likely distributed and abundant in the areas northward of the Polar Circle in Chukotka, but there are currently no available materials to test this suggestion. The intermediate position (with respect to geography) between the two aforementioned taxa is occupied by *B. pseudominor,* which may be regarded as a northern derivate of *B. minor* characterized by dilated cells in the middle of its leaf segments and epixylous occurrence (contrary to the epilithic occurrence in *B. minor*), albeit with much longer cells in its leaf segments. *Blepharostoma prima*, as estimated in the present account, was nevertheless confirmed in the continental mainland from hemiarctic (Ayan) to cool–temperate (South Primorye) zones and not observed in East Asia, although it was also confirmed in Murmansk Province of the Russian Northwest and Finland.

The most common taxa recognized in East Asia have relatively large and granulate oil bodies. Frey and Stech [37] estimated Blepharostomataceae as the sister group to Trichocoleaceae. Trichocoleaceae are characterized by having relatively large (2–4 times larger than in *Blepharostoma*) and finely granulate oil bodies. This may help confirm that the numerous small homogenous-to-obsolete oil bodies among *Blepharostoma* are an apomorphic feature now present in *B. prima*.

## 5. Taxonomic Treatment

***Blepharostoma*** (Dumort.) Dumort., Recueil Observ. Jungerm.: 18, 1835.

= *Jungermannia* sect. *Blepharostoma* Dumort., Syll. Jungerm. Europ.: 65, 1831.

### 5.1. The Key

The tentative key for the taxa of the “*B. trichophyllum* s.l.” complex is as follows:

1. The middle cells in the cilia with length/width ratio less 1.5 … 2

1. The middle cells in the cilia with length/width ratio more 1.5 … 3

2. Segments not dilated, plants from boreal to arctic zones … 3. *B. brevirete* + 8. hybrid taxon 1

2. Segments distinctly dilated, plants from cool temperate to warm temperate zones … 5. *B. minor*

3. Oil bodies as droplets, or small and homogenous, spherical to fusiform, more than 8–10 per cell … 4

3. Oil bodies granulate, spherical to shortly ellipsoidal, less 8 per cell … 5

4. The middle cells in the cilia less 50 μm in length, length/width ratio less 2.8, distributed in hemiarctic to cool-temperate communities … 1. *B. prima*

4. The middle cells in the cilia commonly longer than 50 μm in length, length/width ratio ca 3.3, known only in hemiboreal communities (chiefly epilithic) in the contact zone between East Asian and Circumboreal floras … 2 *B. pseudominor*

5. Leaf cilia segments length/width ratio 1.5-1.8, oil bodies 3–7 per cell warm temperate taxon known from Japan and Korean Peninsula … 4. *B. epilithica*

5. Leaf cilia segments length/width ratio 1.4–2.6 with 3–4 oil bodies or length/width ratio (1.3-)2.0-3.0 with 3-8 oil bodies, hemiarctic to oro-boreal distribution (in China mainland to Yunnan and Sichuan Provinces), not known in Japan and Korea … 6

6. Cilia segments with length/width ratio ca 1.5–1.8, segments not dilated, stem cross Section 6–7 cells high, oil bodies in cilia mostly 3–4 per cell … 7. *B. trichophyllum*

6. Cilia segments with length/width ratio 1.4–2.6, segments dilated, stem cross Section 5 cells high, oil bodies in cilia mostly 3–8 per cell … 9. *B. neglecta*

### 5.2. Descriptions

#### 5.2.1. *Blepharostoma prima* Vilnet et Bakalin sp. nov.

Description. Plants (250–)300–500 μm wide. Stem cross section nearly rounded, 80 μm in diameter, merely rigid, 4 cells high, external wall to 5 μm thick, inward cell walls become thinner, although still thick, trigones vestigial to small and concave, cell size nearly equal across the section, 12–20 μm in diameter. Leaves slightly appressed to the stem (feature—a subject of the great variation), in general view straight at almost whole length, leaf segments in the slide straight to slightly falcate, 350–400 μm long. Middle cells in the segment 25–50(−70) × 16–22 μm, sometimes shorter in gemmiparous plants (20–25 × 18–20 μm), length/width ratio (1.1–)1.4–2.8(−3.5), cells not or slightly dilated; oil bodies homogenous, as droplets, small spherules 1.0–1.5 μm in diameter or shortly fusiform 1.0–1.5 × 1.0–2.0 μm.

Holotype: Russia, Sakhalin Province, Sakhalin Island, Dolinsky District, Anna River Valley in the area adjacent to its mouth (47°09′45.7” N 143°01′43.9” E), 10–50 m alt., coniferous (*Abies sachalinensis* (F. Schmidt) Mast. dominating) forests on steep slope with many rocky outcrops, on moist cliff near stream in open area. Leg. V.A. Bakalin and K.G. Klimova 29 September 2016 S-45-12-16 (VBGI, isotype in KPABG)). The other specimens examined are shown in Table 1.

Comments: *Blepharostoma prima*, in its narrow sense, is characterized by (1) droplet-like oil bodies or oil bodies that are virtually absent, (2) nearly straight outer lines of the segments (not dilated cells), and relatively long (or short in gemmae producing plants) segments. (3) In a stereoscopic (under a dissecting microscope) view, the species is characterized by segments that are nearly entirely straight and sometimes even appressed to the stem, whereas in other locally recognized taxa, the leaf segments are primarily erect and spread for 1/3–2/5 of the length; then, they suddenly curve subparallel to the stem (this feature is subject to great variation). *Blepharostoma prima* is not common in East Asia and known in relatively few localities. This species is malleable in its ecology and known in both epilithic and epixylous habitats. Based on the data in hand, this species sparsely occurs in the southern half of the Russian Far East and northward to 56 °N (Ayan in the Sea of Okhotsk coast). The parameters of variation in the description are likely underestimated due to a limited number of molecularly confirmed specimens.

**Figure 3:**
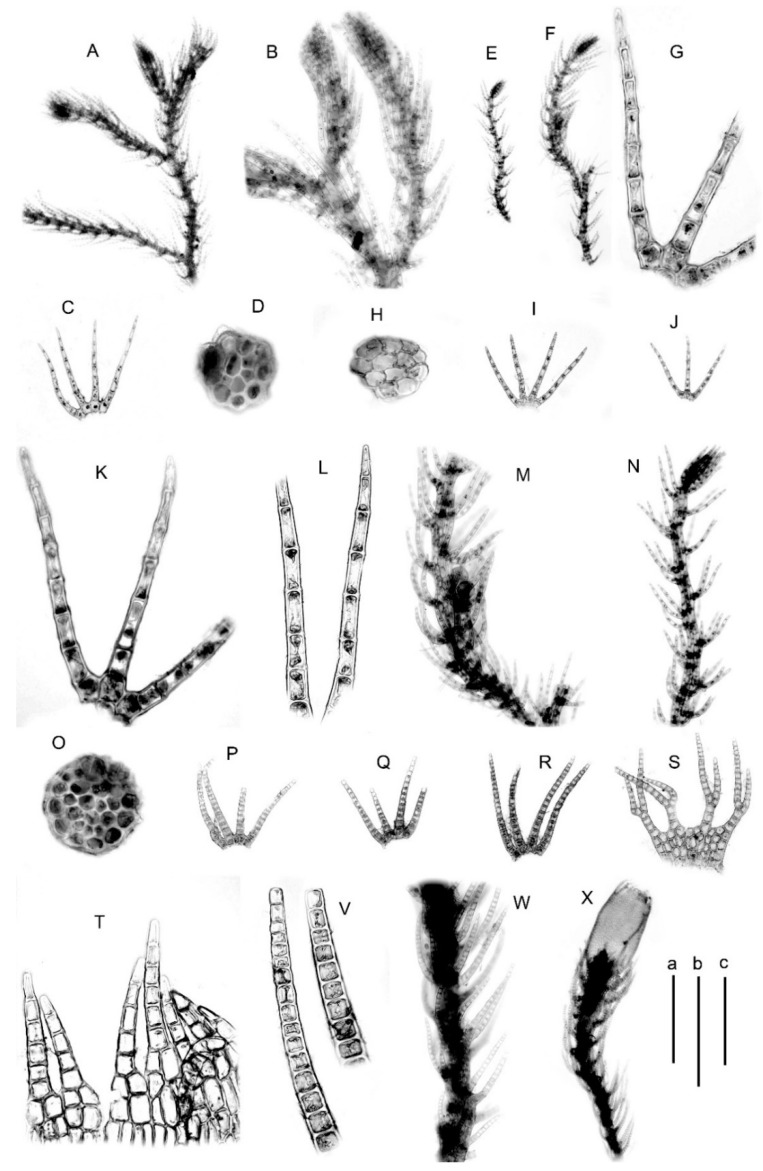
(**A**–**D**) *Blepharostoma prima*, from S-45-12-16 (VBGI); (**E**–**N**) *B. pseudominor*, from P-4-1-08 (VBGI); (**O**–**X**) *B. brevirete*, from Mag-10-1-10 (VBGI). (**A**,**B**,**E**,**F**,**M**,**N**,**W**,**X**)—plant habit; (**C**,**I**,**J**,**P**–**R**)—leaves; (**D**,**H**,**O**)—stem cross-section; (**G**,**K**,**L**,**V**)—leaf segments; (**S**)—female bract; (**T**)—perianth mouth armature. Scales: a—1 mm, for (**A**,**E**,**F**,**X**); b—500 μm, for (**B**,**C**,**I**,**J**,**P**–**S**,**W**); c—100 μm, for (**G**,**D**,**H**,**K**,**L**,**O**,**T**,**V**).

Illustrations in the present paper: Figure 3A–D, Figures S1A and S2A–D.

#### 5.2.2. *Blepharostoma pseudominor* Vilnet et Bakalin sp. nov.

Description. Plants 250–450 μm wide. Stem cross section slightly transversely elliptic, ca (50–)70 × 80–90 μm, very soft, 4 cells high, cells thin-walled (including external wall, or external wall slightly thickened), trigones small, concave, cell size nearly equal in size across section, 15–20 μm in diameter. Leaves commonly 3-lobed, in the slide leaf segments nearly straight, 250–300 μm long. Middle cells in the segment 35–60 × 12–18 μm, length/width ratio ca 3.3, cells dilated; oil bodies homogenous, as droplets, small spherules 1.0–1.5 μm in diameter to shortly fusiform 1.0–1.5 × 1.5–2.5 μm.

Holotype: Russia, Primorye Territory, Livadiysky Range, Pidan Mt. Area, Oyry Stream valley (43 05′05.1” N 132 41′40.3” E), 699 m alt., mountain lighted dark coniferous forest. Leg. V.A. Bakalin 07 October 2008 P-56-15-08 (VBGI, isotype in KPABG). Other specimens examined are in Table 1.

Comment. This is a narrowly distributed species whose area is chiefly restricted to the contact zone of East Asian and Circumboreal Floristic Regions and morphologically resembles *Blepharostoma minor*, although with distinctly longer segments (not characteristic for *B. minor*) and numerous homogenous oil bodies. This species’ very soft stems with thin cell walls in the cross-section, height of only four cells, and distinctly dilated cells in the segment middle are different from *B. prima*. This species is characterized by epixylous ecology, thin segments of moderate length (200–350 μm), slightly dilated leaf segment cells, and numerous and smooth oil bodies. Currently, this species is known in the southern part of Primorsky Territory and Kunashir Island in the southern Kurils.

Illustrations in the present paper: Figure 3E–N.

#### 5.2.3. *Blepharostoma brevirete* (Bryhn et Kaal.) Vilnet et Bakalin comb. nov. Basionym: *Blepharostoma trichophyllum* var. *brevirete* Bryhn et Kaal., Rep. Second Norweg. Arctic Exped. 11: 46, 1906. Syntypes: Canada. King Oskar Land, 76” 30′ N; Ellesmere Island, Framshavn (O, not seen).

Description. Plants 300–600 μm wide, slightly wider near the perianth. Stem cross section slightly transversely elliptic, 6 cells high, ca 100 × 110 μm, external wall to 5 μm thick, inward cell walls thin to thickened, with concave to loosely convex, large, sometimes confluent trigones, cells nearly equal in size across section, 10–17 μm in dimeter, outer surface of the stem striolate. Leaves with segments (220–)350–500 μm long, slightly falcate in the slide. Middle cells in the segment 12–35 × 20–30 μm, length/width ratio ca 1.0–1.8, cells not dilated; oil bodies granulate, 5–8 per cell, spherical 2.5–4.0 μm in diameter to shortly oblong 2.5–4.0 × 3–6 μm. Female bracts with lamina 3–4 cells high, primary segments bifurcate, in general outlook bracts ca 550 × 700 μm. Perianth tubular, not contracted to the mouth, loosely 3–4–plicate (mostly very indistinctly), ca 1000 × (400–)500 μm, mouth ciliate–laciniate, 1–2 cells in the base and 4–7 cells long, with 4–6-celled uniseriate part.

Comment. This taxon is well-defined both morphologically and geographically. It is restricted to Hemiarctic and Arctic vegetation zones, with the southernmost area reported in Pacific Asia from the Eastern Kamchatka. It is characterized by relatively thick and long (300–500 μm) segments, with non-dilated cells and granulate oil bodies (few per cell). However, this taxon is indistinguishable from the *B. trichophyllum* hybrid 1 (as shown in the key and the relevant discussion).

The examined specimens are shown in Table 1.

**Figure 4:**
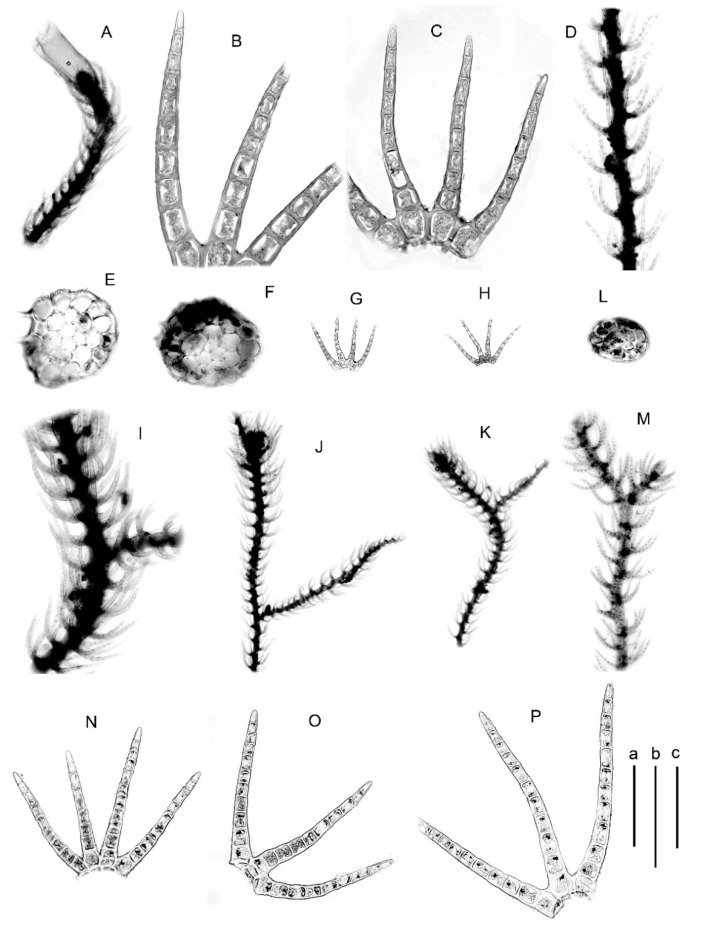
(**A**) *Blepharostoma brevirete*, from Mag-10-1-10 (VBGI); (**B**–**K**) *B. epilithica*, from J-11-13-15 (VBGI); (**L**–**P**) *B. minor*, from J-91-51-15 (VBGI). (**A**,**D**,**I**–**M**)—plant habit; (**B**,**C**)—leaf segments; (**E**,**F**,**L**)—stem cross-section; (**G**,**H**,**N**–**P**)—leaves. Scales: a—1 mm, for (**A**,**J**,**K**); b—500 μm, for (**D**,**G**,**H**,**I**,**M**); c—100 μm, for (**B**,**C**,**E**,**F**,**L**,**M**–**P**).

Illustrations in the present paper: Figure 3O–X, Figure 4A, Figures S1B–D and S2E–H.

#### 5.2.4. *Blepharostoma epilithica* Vilnet et Bakalin sp. nov.

Description. Plants 350–600 μm wide, freely branched. Stem cross section nearly orbicular (5)6 cells high, 70–110 μm in diameter, external wall to 5 μm thick, outer surface striolate, outer cells larger, 15–30 μm in diameter, cells inward 12–18 μm in diameter, thin–walled, trigones small to moderate. Leaf segments 180–280 μm long, straight to slightly falcate in the slide. Middle cells in the segment 22–40 × 15–25 μm, length/width ratio ca 1.5–1.8, cells not or slightly dilated; oil bodies granulate, 3–7 per cell, spherical, 2–4 μm in diameter, to shortly oblong 2.5–4.0 × 3–6 μm, sometimes with the admixture of oil droplets.

Holotype: Japan, Shikoku Island, Kochi Prefecture, Nagaoka-gun, Otoyopcho, Ou, Shiofuri Falls (33 48′09” N 133 41′15” E), 500 m alt. Broadleaved evergreen and deciduous forest in stream valley; moist cliff in part shade. Leg. V.A. Bakalin 25 March 2015 J-11-13-15 (VBGI, isotype in KPABG). Other specimens examined are in Table 1.

Comment. This taxon is known from only three specimens from Japan (Shikoku) and South Korea. This taxon is similar to *Blepharostoma minor* due to its short cilia (120–300 μm) and epilithic habitat. The two species distinctly differ in size (350–600 μm wide in *B. epilithica* versus 250–350 μm in *B. minor*) and stem thickness (6 cells versus 4 cells high), with comparatively longer cells in the segment middle (length/width ratio more than 1.5 versus less than 1.4 in *B. minor*) and longer segment cells. The listed features look as environmentally induced; however, here they were shown to be correlated with genetic differences. The biggest problem is how to distinguish species from *B. neglecta* due to transitions in many features (differences listed in the key) and the distribution in the warm temperate vegetation zones of Japan and Korean Peninsula. Moreover, this warm temperate taxon, however, may be distributed wider that it is now known.

Illustrations in the present paper: Figure 4B–K, Figures S1E and S2I.

#### 5.2.5. *Blepharostoma minor* Horik., Hikobia 1 (2): 104, 1951 [1952] Type: Japan, Settsu [Hyōgo] Pref. Toyono-gun, Mino Mt., Y. Horikawa, 5138, 23 November 1948, Holotype HIRO s.n.!

Description. Plants 250–350 μm wide. Stem cross Section 4 cells high, transversely elliptic, ca 50 × 70 μm, outer wall to 3 μm thick, with striolate surface, inward cells loosely thick–walled, trigones large, concave, sometimes confluent, cells of nearly equal size across section, 10–18 μm in dimeter. Leaf segments 120–150(−250) μm long, falcate in the slide. Middle cells in the segment 12–25 × 8–15 μm, length/width ratio ca 1.25–1.4, cells strongly to slightly (almost none, varying on the same plant, also including the type) dilated; oil bodies granulate, 4–6 per cell, spherical, 2 μm in diameter, to shortly oblong 2 × 2.5–3.0 μm.

Comment. This species is characterized by relatively short and thick to thin, 100–150(−250) μm long segments with a few granulate oil bodies and obligate epilithic ecology. Based on the data in hand, this species is widely distributed in East Asia, from southern Kurils (Kunashir and Shikotan Islands) through to Japan and Korea up to North Vietnam and should be common in China.

The specimens examined are shown in Table 1.

**Figure 5:**
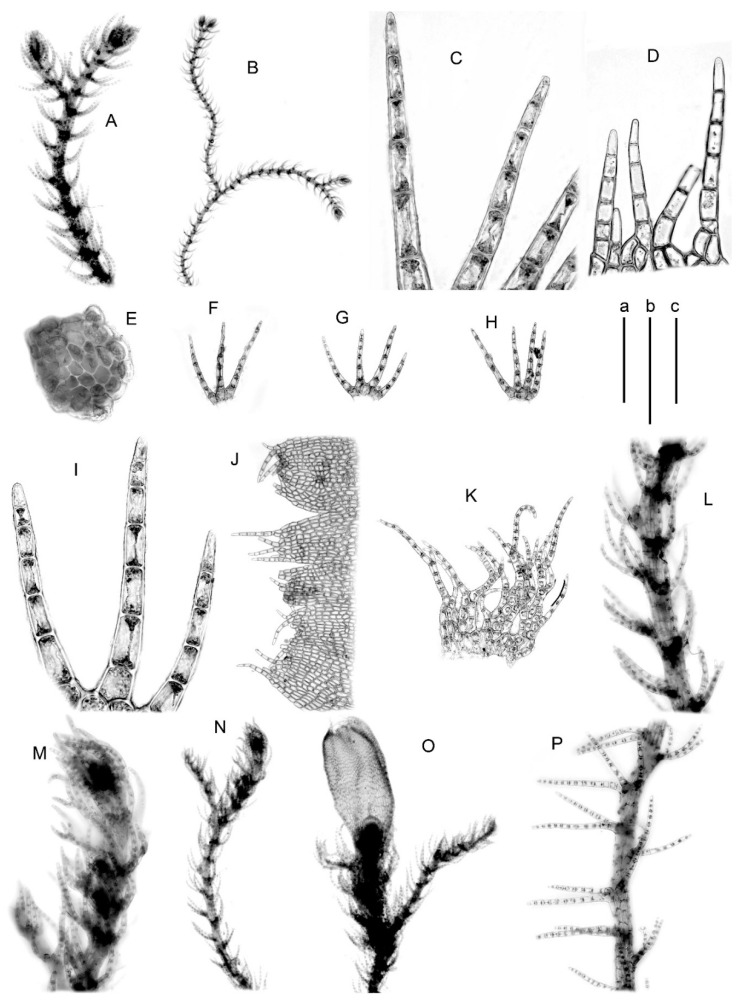
(**A**,**B**) *Blepharostoma minor*, from J-91-51-15 (VBGI); (**C**–**O**) *B. trichophyllum*, from K-43-16-15 (VBGI); (**P**) *B. trichophyllum x hybrid1*, from Mag-57-2-11 (VBGI). (**A**,**B**,**L**–**P**) plant habit; (**C**,**I**)—leaf segments; (**D**,**J**)—perianth mouth armature; (**E**)—stem cross-section; (**F**–**H**)—leaves; (**K**)—female bract. Scales: a—1 mm, for (**B**,**N**,**O**); b—500 μm, for (**A**,**F**–**H**,**J**–**M**,**P**); c—100 μm, for (**C**–**E**).

Illustrations in the present paper: Figure 4L–P, Figure 5A,B and Figrue S2J,K.

#### 5.2.6. Cryptic Taxon (Tentatively Named ‘*B. sp.*’ in the Phylogenetic Tree)

This prospective new taxon is known based on a single specimen, which was not investigated in its living conditions, so its oil bodies’ parameters are not known. Moreover, since it is known from only one specimen, we do not sufficiently understand the morphological variation of the taxon. Therefore, we cannot identify its morphological similarities with other taxa in the *Blepharostoma trichophyllum* complex nor ignore the obtained information. We classify this taxon as a cryptic taxon, until new data on the taxon’s distribution and morphology become available. The plants in the specimen are characterized by thick and long cilia, as well as dilated or non-dilated cells in the middle of the segment. This taxon is similar to *B. brevirete* in both its morphology and ecology (occurring over *Sphagnum* mats in mountainous tundras).

The upper clades on both trees (Figure 1 and Figure 2) feature a series of widely distributed taxa with two of them having a hybrid nature. As noted in the results, the original (ancestral) taxon (*B. trichophyllum*) intermingled with one of the two descendant taxa in each tree. This taxon hybridized (as the maternal parent via cytoplasmic inheritance; blue color in Figure 1 and Figure 2) with one unknown species and developed into the clade ”hybrid 2”. The same ancestral taxon hybridized with another unknown species (as the ‘father’ via nuclear inheritance; red color), which resulted in the clade hybrid 1. The hybrid 1 clade is morphologically dissimilar to its ‘father’, *B. trichophyllum,* and strongly resembles *B. brevirete* (although not related molecularly), whereas hybrid 2 (cytoplasmic inheritance of *B. trichophyllum*) possesses some morphological features of its ‘mother’ taxon, *B. trichophyllum*. However, the hybridization in this group was a very old event. Now, both hybrids are widely distributed and stable in a molecular-genetic and morphological sense. Therefore, the two hybrid taxa may be regarded as independent species, despite having an initially hybrid nature. Here, we prefer to regard one of the hybrids (hybrid 1, which is morphologically similar to *B. brevirete*) as the cryptic species and refrain from describing it properly in nomenclatural terms. However, hybrid 2 can be well-circumscribed morphologically, so we provide it with the name *Blepharostoma neglecta* sp. nov.

#### 5.2.7. *Blepharostoma trichophyllum* (L.) Dumort. (Green Colored Clade in the Figure 1 and Figure 2) Lectotype: United Kingdom,”, in Sylva Gleibergensi, Loco Fundusniger Dicto” (OXF, Not Seen)

Description. Plants 300–500 μm wide. Stem cross section nearly rounded, 6–7 cells high, 90–120 μm in diameter, outer cells distinctly larger than inner, 20–30 μm in diameter, external wall to 5 μm thick, outer surface striolate, inner cells loosely thickened, with small, concave to triangle trigones, 12–22 μm in diameter. Leaf segments 220–350 μm long, straight to slightly falcate in the slide. Middle cells in the segment 30–70 × 20–28 μm, length/width ratio 1.4–2.6, cells not dilated; oil bodies granulate, 3–4(−10) per cell, spherical 2–3 μm in diameter, to shortly oblong 2–3 × 3–5 μm. Male bracts with cilia commonly bifurcate. Female bracts with lamina 2 cells high, lobes several times bifurcate (at maximum with 6 cilia of the second order on one ‘lobe’), terminal cilia variously curved. Perianth loosely 3–4–plicate in upper 2/3, slightly obovate, distinctly or loosely contracted to the mouth, ca 1300 × 700 μm, mouth ciliate, cilia 4–6 cells long, sometimes from 2-celled base. Elaters 210–300 × 10–11 μm; spores 15–18 μm in diameter.

Comment. This species is characterized by oblong and relatively narrow, not dilated cells in the middle part of the segment and a few granulate oil bodies. Based on the data in hand, this species is widely distributed northward of the middle Sakhalin Island (50 °N) to the middle part of the Kamchatka Peninsula and Magadan Province (to 60 °N). Moreover, this taxon is not rare outside of the Russian Far East and was recorded in other areas of the Hemiarctic and southward in the mountains, including the following (these data are surely incomplete, as they are based only on genetically confirmed identifications): the Murmansk Province of European Russia, Wyoming in the USA, British Columbia in Canada, and possibly in Germany (only trnL data).

The original characteristics of the oil bodies in *Blepharostoma trichophyllum* provided in Schuster [1] for North America and Damsholt [2] and Paton [3] for European countries include the indication of few granulate oil bodies. Schljakov [4] was the first to observe numerous and very small homogenous oil bodies in *B. trichophyllum*, along with a few granulate oil bodies in the same species. Here, the most common taxon, *B. trichophyllum* s. str., which is known to exist in various parts of the Holarctic, is characterized by a few granulate oil bodies, whereas the ‘phases’ with numerous and homogenous oil bodies belong to *B. prima* and *B. pseudominor*.

The specimens examined are shown in Table 1.

Illustrations in the present paper: Figure 5C–O, Figures S1F–H and S2L–R.

#### 5.2.8. *Blepharostoma trichophyllum* Hybrid Taxon 1 (Red Colored Clade in Figure 1 and Figure 2)

Description. Plants 500–800 μm wide. Stem cross section slightly transversely elliptic, 4–5 cells high, ca 110 × 90 μm, to rounded, 90–100 μm in diameter, external wall to 5 μm thick, with surface loosely striolate, cells nearly equal in size across section, (12–)15–30 μm in diameter, outer layer with large concave trigones, inward trigones become small. Leaves 4-lobed, but also commonly 3-lobed (3–ciliate). Leaf segments slightly falcate to nearly straight, 300–420 μm long. Middle cells in the leaf segment 20–40 × 22–30 μm, length/width ratio ca 0.9–1.3, cells not dilated, with striolate surface; oil bodies granulate, 3–6 per cell, spherical 3–5 μm in diameter to shortly oblong 2.5–3.0 × 3–5 μm.

Comment. This taxon is very similar to *Blepharostoma brevirete* due to its short cells in the middle of the leaf segments (however, sometimes these cells are longer than those in typical *B. brevirete*). The segments are somewhat shorter than those in *B. brevirete*, but this length parameter in the two taxa is connected by a series of transitional forms (even within the same patch), which represent two extremes. This taxon seems to be indistinctly more southerly distributed and restricted to hemiarctic and boreal zones, although it was also observed in Spitsbergen and, therefore, cannot be distinguished from *B. brevirete* on a geographical basis with confidence.

**Figure 6:**
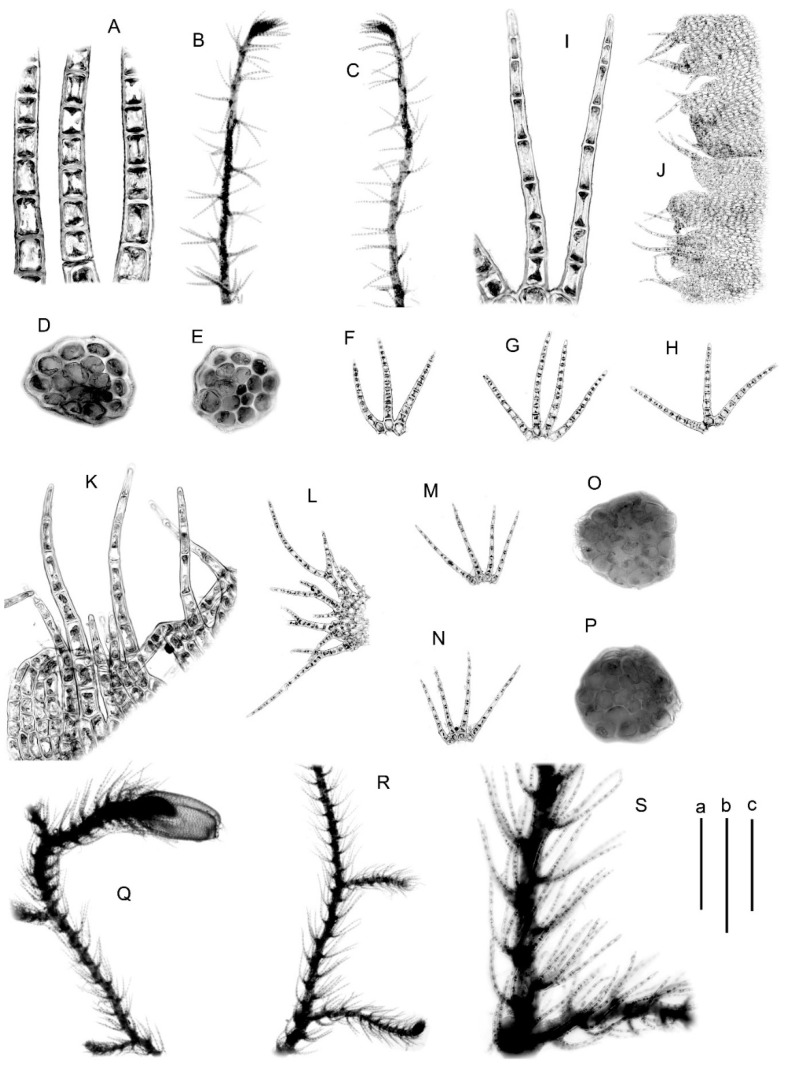
(**A**–**H**) *Blepharostoma trichophyllum x hybrid1*, from Mag-57-2-11 (VBGI); (**I**–**S**) *B. neglecta*, from S-48-18-16 (VBGI). (**A**,**I**)—leaf segments; (**B**,**C**,**Q**–**S**)—plant habit; (**J**,**K**)—perianth mouth armature; (**D**,**E**,**O**,**P**)—stem cross-section; (**F**–**H**,**M**,**N**)—leaves; (**L**)—female bract. Scales: a—1 mm, for (**B**,**C**,**Q**,**R**); b—500 μm, for (**F**–**H**,**J**,**L**,**N**,**S**); c—100 μm, for (**D**,**E**,**K**,**O**,**P**).

Illustrations in the present paper: Figure 5P, Figure 6A–H, Figrues S1I,J and S2S,T.

#### 5.2.9. *Blepharostoma neglecta* Vilnet et Bakalin sp. nov. (Hybrid Taxon 2, Blue Colored in the Figure 1 and Figure 2)

Description. Plants 500–700 μm wide. Stem cross section nearly rounded to transversely elliptic, 5–6 cells high, 90–100 μm in diameter, to 90 × 110 μm, outer cells slightly larger than inner, 20–25(−30) μm in diameter, external wall 1–2 μm thick, outer surface smooth to striolate, trigones adjacent to external wall large and concave, inward become moderate to small in size, cells in inner layer 15–22 μm in diameter. Leaf segments 300–380 μm long, nearly straight in the slide. Middle cells in the segment (20)30–45(55) × 12–20 μm, length/width ratio ca (1.3–)2.0–3.0, cells dilated; oil bodies granulate, 3–8 per cell, spherical 2–3 μm in diameter to shortly oblong 2–3 × 3–4 μm, sometimes with admixture of droplets. Female bracts in general outlook 500 × 800 μm, with lamina 3 cells high with additional lateral cilia, main ‘lobes’ unequally bifurcate. Perianth ovate, loosely 3-plicate, 900–1000 × 500 μm, contracted to the mouth, mouth ciliate, cilia 4–6-celled, composed by strongly elongate cells, 30–40 × 10–20 μm. Elaters 200–2500 × 10–12 μm; spores 10–13 μm in diameter.

Holotype: Russia, Sakhalin Province, Sakhalin Island middle part, Zhdanko Mt. (48 05′27.6” N 142 31′30.2” E), 190–350 m alt. Coniferous forest on steep rocky slope in stream valley, with dense *Taxus cuspidata* Sieb. et Zucc. ex Endl. understory; moist boulder near stream, in part shade. Leg. V.A. Bakalin 1 October 2016 S-48-18-16 (VBGI, isotype in KPABG). Other specimens examined are in Table 1.

Comment. This taxon is characterized by narrow leaf segments composed of relatively long and dilated cells; in this respect, it strongly resembles *B. pseudominor*, although it distinctly differs in its granulate oil bodies, which are few in number. On the other hand, this taxon is morphologically similar to *B. trichophyllum* but differs in having narrower cilia with comparatively longer cells. The difference in its spore size is not clear due to the very limited materials available for comparison. Based on the data in hand, this taxon is mostly distributed in hemiboreal landscapes. Within the Russian Far East, it mostly occurs in the insular-peninsular part of the area, from 56 °N in Central Kamchatka to middle Sakhalin and southern Kurils (48 °N). In the mainland, it is known in the southern part of Magadan Province and Primorsky Territory. Southward, it occurs in the Yunnan and Sichuan Provinces of China and is likely distributed more widely than now known. Westward, the taxon is confirmed in eastern Siberia (Zabaikalsky Territory) and Eastern Europe in the Perm Province and Mary-El Republic of Russia.

Illustrations in the present paper: Figure 6I–S, Figrues S1K,L and S2U–AE.

## Figures and Tables

**Table 1:** The list of taxa with notes on preliminary identifications, specimen vouchers, and GenBank accession numbers. Accession starts from MT were sequenced in the current study.

Species	Preliminary Identification	Country	Specimen Voucher	Collector	GenBank	
					ITS1-2 nrDNA	*trn*L-F cpDNA
*B. arachnoideum*	*arachnoideum*	USA: Oregon	SM-2019-25 (VBGI)	D. Wagner	MT586201	MT585796
*B. arachnoideum*	*arachnoideum*	USA: Oregon	KB18-102 (VBGI)	D. Wagner	MT586202	MT585797
*B. arachnoideum*	*arachnoideum*	USA: Oregon	17421 (VBGI)	D. Wagner	MT586203	MT585798
*B. arachnoideum*	*arachnoideum*	USA: Oregon	JS19-286 (VBGI)	S. Jessup	MT586204	no data
*B. arachnoideum*	*arachnoideum*	USA: Oregon	GV18-1047 (VBGI)	D. Wagner	MT586205	MT585799
*B. brevirete* comb. nov.	*trichophyllum* subsp. *brevirete*	Greenland		K. Hassel, T. Presto	KC333191	no data
*B. brevirete* comb. nov.	*trichophyllum* subsp. *brevirete*	Norway: Svalbard	K-130-4c-04, 110,026 (KPABG)	N. Konstantinova	MT586187	MT585780
*B. brevirete* comb. nov.	*trichophyllum*	Norway: Svalbard	K301-3-11, 115,395 (KPABG)	N. Konstantinova	MT586188	MT585781
*B. brevirete* comb. nov.	*trichophyllum* subsp. *brevirete*	Russia: Kamchatka Prov.	K-50-11-15, 300,157 (VBGI)	V. Bakalin	MT586189	MT585782
*B. brevirete* comb. nov.	*trichophyllum* subsp. *brevirete*	Russia: Magadan Prov.	Mag-10-1-10, 313,792 (VBGI), 115,163 (KPABG)	V. Bakalin	MT586190	MT585783
*B. brevirete* comb. nov.	*trichophyllum*	Russia: Murmansk Prov.	Mur-34-14-15, 308,832 (VBGI)	V. Bakalin	MT586191	MT585784
*B. epilithica* sp. nov.	*minor*	Japan: Kochi	J-11-13-15 (VBGI)	V. Bakalin	MT586185	MT585777
*B. epilithica* sp. nov.	*trichophyllum*	South Korea: Gangwon Prov.	37-10, 115,503 (KPABG)	S.-S. Choi	MT586186	MT585778
*B. epilithica* sp. nov.	*minor*	South Korea: Gangwon Prov.	Kor-11-16-11, 317,266 (VBGI), 115,597 (KPABG)	V. Bakalin	no data	MT585779
*B. minor*	*minor*	Japan: Miyazaki Pref.	J-91-51-15, 303,782 (VBGI)	V. Bakalin	MT586179	MT585770
*B. minor*	*minor*	Russia: Sakhalin Prov., Kuril I., Kunashir Isl.	K-36-13-06, 311,880 (VBGI), 115,146 (KPABG)	V. Bakalin	MT586180	MT585771
*B. minor*	*minor*	Russia: Sakhalin Prov., Kuril I., Shikotan Isl.	K-49-6-07, 313,369 (VBGI), 115,152 (KPABG)	V. Bakalin	MT586181/MT586210	MT585772
*B. minor*	*minor*	South Korea: Jeonbuk Prov.	Kor-14-19-08, 311,767 (VBGI), 115,150 (KPABG)	V. Bakalin	no data	MT585773
*B. minor*	*minor*	South Korea: Jeonbuk Prov.	Kor-17-57-08, 311,857 (VBGI), 115,151 (KPABG)	V. Bakalin	MT586182/MT586211	MT585774
*B. minor*	*minor*	South Korea: Gyeongnam Prov.	Kor-16-19a-09, 317,099 (VBGI), 115,153 (KPABG)	V. Bakalin	MT586183	MT585775
*B. minor*	*minor*	Vietnam: Lao Cai Prov.	V-1-27-17, 35,095 (VBGI), 122,638 (KPABG)	V. Bakalin & K.Klimova	MT586184	MT585776
*B. prima* sp. nov.	*trichophyllum*	Finland		He-Nygren &Piippo	DQ293944	no data
*B. prima* sp. nov.	*trichophyllum*	Russia: Khabarovsk Terr.	kh-47-28-19 (VBGI)	V. Bakalin	MT586195	MT585790
*B. prima* sp. nov.	*trichophyllum*	Russia: Murmansk Prov.	B72-10-10, 20,464 (KPABG)	O. Belkina	MT586196	MT585791
*B. prima* sp. nov.	*minor*	Russia: Primorsky Terr.	G116245 (KPABG)	E. Borovichev	MT586197	MT585792
*B. prima* sp. nov.	aff. *trichophyllum*	Russia: Sakhalin Prov.	S-14-10-17, 122,635 (KPABG)	V. Bakalin	MT586198	MT585793
*B. prima* sp. nov.	*trichophyllum*	Russia: Sakhalin Prov.	S-45-12-16 (VBGI)	V. Bakalin	MT586199	MT585794
*B. prima* sp. nov.	*trichophyllum*	Russia: Sakhalin Prov.	S-49-13-09, 309,815 (VBGI), 115,158 (KPABG)	V. Bakalin	MT586200	MT585795
*B. pseudominor* sp. nov.	*trichophyllum*	Russia: Primorsky Terr.	P-4-1-08, 310,194 (VBGI), 115,159 (KPABG)	V. Bakalin	MT586192	MT585785
*B. pseudominor* sp. nov.		Russia: Primorsky Terr.	P-56-15-08 (VBGI)	V. Bakalin	MT586193	MT585786
*B. pseudominor* sp. nov.	cf. *minor*	Russia: Sakhalin Prov.	S-24-5-06, 312,731 (VBGI), 115,149 (KPABG)	V. Bakalin	no data	MT585787
*B. pseudominor* sp. nov.	*minor*	Russia: Sakhalin Prov., Kuril I., Kunashir Isl.	K-42-1-06, 312,097 (VBGI), 110,259 (KPABG)	V. Bakalin	no data	MT585788
*B. pseudominor* sp. nov.	*minor*	Russia: Sakhalin Prov., Kuril I., Kunashir Isl.	K-44-21-18, 57,923 (VBGI), 122,499 (KPABG)	V. Bakalin, K.Klimova	MT586194	MT585789
Possible cryptic taxon tentatively named as ‘*B. sp.*’	*brevirete*	Russia: Khabarovsk Terr.	Kh-40-31-13, 302,859 (VBGI)	V. Bakalin	MT586178	MT585769
*B. trichophyllum* (“green” parent)	*brevirete*	Russia: Magadan Prov.	Mag-29-26-14, 301,897 (VBGI)	V. Bakalin	MT586135	MT585723
*B. trichophyllum* (“green” parent)	*trichophyllum*	Russia: Magadan Prov.	Mag-34-26-12, 306,377 (VBGI)	V. Bakalin	MT586136	MT585724
*B. trichophyllum* (“green” parent)	*trichophyllum*	Russia: Murmansk Prov.	G20760 (KPABG)	E. Borovichev	MT586137	MT585725
*B. trichophyllum* (“green” parent)	*minus*	Russia: Kamchatka Terr.	K-48-1-15, 300,081 (VBGI)	V. Bakalin	MT586138	MT585726
*B. trichophyllum* (“green” parent)	*brevirete*	Russia: Kamchatka Terr.	K-66-13-15, 300,362 (VBGI)	V. Bakalin	MT586139	MT585727
*B. trichophyllum* (“green” parent)	*trichophyllum*	Russia: Kamchatka Terr.	K-43-16-15, 300,019 (VBGI)	V. Bakalin	MT586140	MT585728
*B. trichophyllum* (“green” parent)	*trichophyllum*	Russia: Khabarovsk Terr.	Kh-46-12-19 (VBGI)	V. Bakalin	MT586141	MT585729
*B. trichophyllum* (“green” parent)	*trichophyllum*	Russia: Khabarovsk Terr.	Kh-50-17-19 (VBGI)	V. Bakalin	MT586142	MT585730
*B. trichophyllum* (“green” parent)	*trichophyllum*					
*B. trichophyllum* (“green” parent)	*trichophyllum* var. brevirete	Russia: Sakhalin Prov.	S-25-8-17, 122,637 (KPABG)	V. Bakalin	MT586143	MT585731
*B. trichophyllum* (“green” parent)	*trichophyllum*	Russia: Sakhalin Prov.	S-30-9-09, 309,613 (VBGI), 115,162 (KPABG)	V. Bakalin	MT586144	MT585732
*B. trichophyllum* (“green” parent)	*trichophyllum*	Russia: Yakutia Rep.	23-5-00, 101,623 (KPABG)	V. Bakalin	MT586145	MT585733
*B. trichophyllum* (“green” parent)	*trichophyllum*	USA: Wyoming	8092 (VBGI)	Kosovich	MT586146	MT585734
*B. trichophyllum* (“green” parent)	*trichophyllum*	USA	ACC#B127596 (KPABG)	Schofield W., R. Belland, T. Hedderson	MT586147	MT585735
*B. trichophyllum* hybrid taxon 1	*trichophyllum* var. *brevirete*	Norway: Svalbard	K-8-1-10, 113,992 (KPABG)	N. Konstantinova, A. Savchenko	MT586168	MT585758
*B. trichophyllum* hybrid taxon 1	*trichophyllum* var. *brevirete*	Norway: Svalbard	12-11-13, 116,486 (KPABG)	N. Koroleva	MT586169/MT586208	MT585759
*B. trichophyllum* hybrid taxon 1	*trichophyllum* var. *brevirete*	Russia: Arkhangelsk Prov., Franz Josef Land, Jekson Isl.	CA19-32-6a	A. Savchenko	MT586148	MT585760
*B. trichophyllum* hybrid taxon 1	*trichophyllum* var. *brevirete*	Russia: Buryatiya Rep.	OAf03108, 113,959 (KPABG)	O. Afonina	MT586170	MT585761
*B. trichophyllum* hybrid taxon 1	*trichophyllum* var. *brevirete*	Russia: Khabarovsk Terr.	Kh-58-4-19 (VBGI)	V. Bakalin	MT586171	MT585762
*B. trichophyllum* hybrid taxon 1	*trichophyllum*	Russia: Komi Rep.	110236 (KPABG)	M. Dulin	MT586172	MT585763
*B. trichophyllum* hybrid taxon 1	*trichophyllum* var. *brevirete*	Russia: Komi Rep.	115075 (KPABG)	M. Dulin	MT586173/MT586209	MT585764
*B. trichophyllum* hybrid taxon 1	*trichophyllum*	Russia: Magadan Prov.	Mag-57-2-11, 316,644 (VBGI),	V. Bakalin	MT586174	MT585765
*B. trichophyllum* hybrid taxon 1	*trichophyllum* var. *brevirete*	Russia: Sakhalin Prov.	S-18-24-17, 122,634 (KPABG)	V. Bakalin	MT586175	MT585766
*B. trichophyllum* hybrid taxon 1	*trichophyllum* var. *brevirete*	Russia: Sakhalin Prov.	S-32-27a-06, 115,166 (KPABG), 313,003 (VBGI)	V. Bakalin	MT586176	MT585767
*B. trichophyllum* hybrid taxon 1	*trichophyllum* var. *brevirete*	Russia: Yakutiya Rep.	WF9880718-15 (KPABG)	Filin	MT586177	MT585768
*B. neglecta* sp. nov. (hybrid taxon 2)	aff. *trichophyllum*	China: Sichuan Prov.	China-44-10-17, 122,632 (KPABG), 37,380 (VBGI)	V. Bakalin, K. Klimova	MT586150	MT585736
*B. neglecta* sp. nov. (hybrid taxon 2)	sp. indet.	China: Yunnan Prov.	C-77-18-18 (VBGI)	V. Bakalin	MT586151	MT585737
*B. neglecta* sp. nov. (hybrid taxon 2)	*trichophyllum*	Norway		H. Blom	KC333189	no data
*B. neglecta* sp. nov. (hybrid taxon 2)	*trichophyllum*	Norway		J. Jordal	KC333190	no data
*B. neglecta* sp. nov. (hybrid taxon 2)	*trichophyllum*	Russia: Kamchatka Terr.	K-133-40-04, 115,167 (KPABG), 309,158 (VBGI)	V. Bakalin	MT586152	no data
*B. neglecta* sp. nov. (hybrid taxon 2)		Russia: Khabarovsk Terr.	Kh-46-3-19 (VBGI)	V. Bakalin	MT586153	MT585738
*B. neglecta* sp. nov. (hybrid taxon 2)	*trichophyllum*	Russia: Krasnodar Terr.	K473-4-07, 111,729 (KPABG)	N. Konstantinova	MT586154	MT585739
*B. neglecta* sp. nov. (hybrid taxon 2)	*trichophyllum*	Russia: Magadan Prov.	Mag-18-4-10, 115,164 (KPABG), 313,978 (VBGI)	V. Bakalin	MT586155	MT585740
*B. neglecta* sp. nov. (hybrid taxon 2)	*trichophyllum*	Russia: Maryi-El Rep.	K422-1-04, 107,997 (KPABG)	N. Konstantinova	MT586156/MT586206	MT585741
*B. neglecta* sp. nov. (hybrid taxon 2)	*trichophyllum*	Russia: Murmansk Prov.	507-5-04, 12,268 (KPABG)	N. Konstantinova	MT586157	no data
*B. neglecta* sp. nov. (hybrid taxon 2)	*trichophyllum*	Russia: Perm Terr.	K354-5-04, 108,337 (KPABG)	N. Konstantinova	MT586158/MT586207	MT585742
*B. neglecta* sp. nov. (hybrid taxon 2)	*minor*	Russia: Primorsky Terr.	P-56-10-08, 115,148 (KPABG)	V. Bakalin	MT586159	MT585743
*B. neglecta* sp. nov. (hybrid taxon 2)	*sp.*	Russia: Primorsky Terr.	Prim-16-22-16 (VBGI)	V. Bakalin	MT586160	MT585744
*B. neglecta* sp. nov. (hybrid taxon 2)	aff. *trichophyllum*	Russia: Sakhalin Prov.	S-14-7-17, 122,636 (RPABG)	V. Bakalin	MT586161	MT585745
*B. neglecta* sp. nov. (hybrid taxon 2)	*minor*	Russia: Sakhalin Prov.	S-27-8-09, 115,154 (KPABG), 309,566 (VBGI)	V. Bakalin	MT586162	MT585746
*B. neglecta* sp. nov. (hybrid taxon 2)	cf. *minus*	Russia: Sakhalin Prov.	S-48-18-16 (VBGI)	V. Bakalin	MT586163	MT585747
*B. neglecta* sp. nov. (hybrid taxon 2)	*trichophyllum*	Russia: Sakhalin Prov.	S-59-9-09, 309,959 (VBGI), 115,156 (KPABG)	V. Bakalin	MT586164	MT585748
*B. neglecta* sp. nov. (hybrid taxon 2)	*trichophyllum*	Russia: Sakhalin Prov., Kuril I., Iturup Isl.	K-32-15-07, 311,588 (VBGI), 115,160 (KPABG)	V. Bakalin	MT586165	MT585749
*B. neglecta* sp. nov. (hybrid taxon 2)	*minor*	Russia: Sakhalin Prov., Kuril I., Iturup Isl.	K-71-32-15, 308,208 (VBGI)	V. Bakalin	MT586166	MT585751
*B. neglecta* sp. nov. (hybrid taxon 2)	*trichophyllum*	Russia: Trans-Baikal Terr.	YuM95-2-8-11, 115,321 (KPABG)	Yu. Mamontov	MT586167	MT585752
*B. trichophyllum* unclear taxonomic position *	*trichophyllum* subsp. *brevirete*	Norway		K. Hassel, T. Presto	KC333193	no data
*B. trichophyllum* unclear taxonomic position *	*trichophyllum* subsp. *brevirete*	Greenland		K. Hassel, T. Presto	KC333192	no data
*B. trichophyllum* unclear taxonomic position *	*trichophyllum*	Russia: Magadan Prov.	Mag-8-20-10, 313,747 (VBGI), 115,169 (KPABG)	V. Bakalin	MT586149	no data
*B. trichophyllum* unclear taxonomic position *	cf. *minor*	Russia: Sakhalin Prov., Kuril I., Kunashir Isl.	K-34-19-18, 57,099 (VBGI), 122,498 (KPABG)	V. Bakalin, K.Klimova	no data	MT585750
*B. trichophyllum* unclear taxonomic position *	*trichophyllum*	Germany	A30915 (KPABG)	no data	no data	MT585753
*B. trichophyllum* unclear taxonomic position *	*trichophyllum*	Russia: Kemerovo Prov.	18-6-00, 101,790 (KPABG)	N. Konstantinova	no data	MT585754
*B. trichophyllum* unclear taxonomic position *	*Trichophyllum*	Russia: Khabarovsk Terr.	Kh-5-46-07, 115,161 (KPABG)	V. Bakalin	no data	MT585755
*B. trichophyllum* unclear taxonomic position *	*Trichophyllum*	Russia: Sakhalin Prov.	S-28-4-17, 122,633 (KPABG)	V. Bakalin	no data	MT585756
*B. trichophyllum* unclear taxonomic position *	*Trichophyllum*	Russia: Sakhalin Prov.	S-30-7-09, 309,611 (VBGI), 115,168 (KPABG)	V. Bakalin	no data	MT585757

* the position cannot be identified with confidence since data on some genes is absent.

**Table 2:** The list of taxa with specimen vouchers and GenBank accession numbers for the outgroup.

Species	Country	Specimen Voucher	Collector	GenBank	
				ITS1-2 nrDNA	*trn*L-F cpDNA
*Archeophylla schusteri* (E.A.Hodgs. et Allison) R.M.Schust.				no data	FJ173295
*Chiloscyphus coadunatus* (Sw.) J.J. Engel & R.M. Schust.	Jamaica	35035 (JE)	Schaefer-Verwimp & Verwimp	KT992510	no data
*Herbertus sakuraii* (Warnst.) S. Hatt.	Russia	P-74-15-05 (KPABG)	V. Bakalin	EU791849	EU791724
*Hygrobiella nishimurae* N. Kitag.	Japan	ExJ-11-42-15 (VBGI, KPABG)	V. Bakalin	MH580594	MH580592
*Leiomitra lanata* (Hook.) R.M.Schust.	New Zealand	8521	D. Glenny	no data	AY463565
*Lepidozia obtusiloba* Steph.	Australia	NSW 110	E. Cooper	JX289187	JX289513
*Mastigophora woodsii* (Hook.) Nees	China	33696 (E)	D. Long	no data	JF316581
	Scotland		D. Long	JN113529	no data
*Plagiochila sikhotensis* Bakalin & Vilnet	Russia		V. Bakalin	MF947695	MF947697
*Pseudolepicolea quadrilaciniata* (Sull.) Fulford et J.Taylor.	Argentina	31658 (E)	D. Long	no data	KJ802069
*Telaranea neesii* (Lindenb.) Fulford	Borneo	NSW	E. Brown	no data	JX289464
*Temnoma pulchellum* (Hook.) Mitt.	New Zealand	92/87 (F)	J.Braggins	no data	JF316575
*Trichocolea tomentosa* (Sw.) Gottsche	Ecuador	368 (DUKE)	K. Davis	no data	AY608144
*Trichocolea tomentella* (Ehrh.) Dumort.	China	1137	He-Nygren	no data	AY463590
*Trichocolea tomentella* (Ehrh.) Dumort.				no data	AY456277
*Vetaforma dusenii* (Steph.) Fulford et J.Taylor	Chile	11423 (H)	J.J. Engel	DQ293965	AY463593

**Table 3:** The value of the ITS1-2/*trn*L-F *p*-distances for the genus *Blepharostoma*, n/c—non-calculated value due to a single studied specimen.

Number	Taxon	Infraspecific *P*-Distances, ITS1-2/*trn*L-F, %	Infrageneric *P*-Distances, ITS1-2/*trn*L-F, %									
			1	2	3	4	5	6	7	8	9	10
1	*B. trichophyllum*	0.9/0.4										
2	*B. neglecta* sp. nov. hybrid 2	1.4/1.1	8.1/0.9									
3	*B. trichophyllum* hybrid 1	1.2/0.2	1.4/2.6	8.3/3.2								
4	*B. ‘sp’* cryptic taxon	n/c/n/c	6.9/3.3	6.7/3.9	7.1/2.7							
5	*B. minor*	2.0/0.4	10.5/3.8	10.4/4.4	10.6/3.5	9.4/3.6						
6	*B. epilytica* sp. nov.	0.6/0.6	7.5/3.2	7.3/3.7	7.8/3.2	6.8/3.3	9.1/3.5					
7	*B. brevirete* comb. nov.	0.0/0.5	6.9/6.8	6.6/7.4	7.0/6.4	5.7/5.4	8.4/7.2	5.8/7.4				
8	*B. pseudominor* sp. nov.	0.2/0.5	10.3/8.3	9.4/9.0	10.3/7.7	9.4/6.8	11.2/7.9	10.2/8.8	7.7/3.3			
9	*B. prima* sp. nov.	0.2/0.5	16.6/12.7	16.6/13.4	16.7/13.1	17.2/11.2	18.5/12.9	16.3/12.8	15.3/8.8	16.6/10.3		
10	*B. arachnoideum*	0.0/0.3	20.0/11.5	19.3/12.3	19.6/11.9	20.1/9.9	20.7/12.2	20.1/13.1	18.5/7.7	18.6/9.8	21.9/9.2	

## Supplementary Materials

The following are available online at https://www.mdpi.com/2223-7747/9/11/1423/s1, Figure S1: (A) *Blepharostoma prima* (Kh-47-28-19, VBGI); (B–D) *B. brevirete* (B, C from Mur-34-14-15, D from K-50-11-15, all in VBGI); (E) *B. epilithica* (J-11-13-15); (F–H) *B. trichophyllum* (F from S-25-18-17, G from S-25-8-17, H from Kh-50-17-19, all in VBGI); (I,J) *B. trichophyllum x hybrid1* (I from S-18-24-17, J from Kh-58-4-19); (K,L) *B. neglecta* (K from Prim-16-22-16, L from S-48-18-16, all in VBGI)., Figure S2: (A–D) *Blepharostoma prima* (A from S-45-12-16, B, C from Kh-47-28-19, D from S-14-17, all in VBGI); (E–H) *B. brevirete* (E, F from Mur-34-14-15, G, H – from K-50-11-15, all in VBGI); (I) *B. epixylica* (from J-11-13-15, VBGI); (J,K) *B. minor* (J from V-1-27-17, K from J-91-51-15, all in VBGI); (L–R) *B. trichophyllum* (L–N from Kh-46-12-19, O, P from Kh-50-17-19, Q, R from S-25-8-17, all in VBGI); (S,T) *B. trichophyllum x hybrid1* (from kh-58-4-19, VBGI); (U–AE) *B. neglecta* (U, AE from Kh-46-3-19, V, W from C-44-10-17, X–Z from Kh-46-3-19, AA from S-48-18-16, AB, AC from S-14-7-17, AD from Prim-16-22-16, all in VBGI). A–L, O–T, V–AD – leaf segments; M, N, U, AE – elaters and spores.
