# Supplementary material for: Blepharostoma trichophyllum S.L. (Marchantiophyta): The Complex of Sibling Species and Hybrids
Source: Plants (Basel). 2020 Oct 23;9(11):1423. doi: 10.3390/plants9111423 (PMC7716226; doi:10.3390/plants9111423)
Supplement: Supplementary file 1 [file plants-09-01423-s001.zip › plants-952305 - supplementary.pdf]

plants

MDPI

Supplementary material

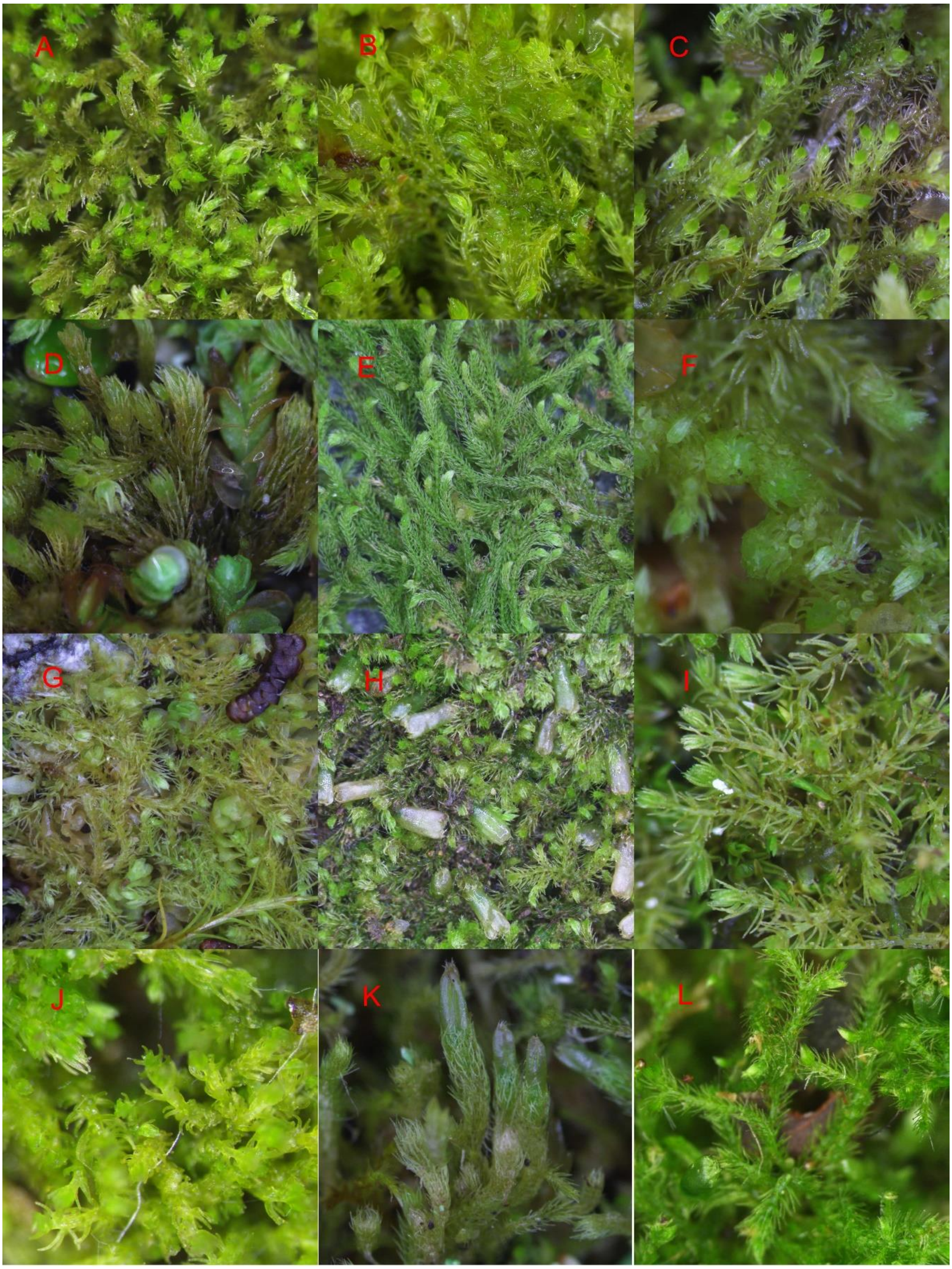


**Figure 1.** (**A**) Blepharostoma prima (Kh-47-28-19, VBGI); (**B–D**) B. brevirete (**B, C** from Mur-34-14-15, **D** from K-50-11-15, all in VBGI); (**E**) B. epilithica (J-11-13-15); (**F–H**) B. trichophyllum (**F** from S-25-18-17, **G** from S-25-8-17, **H** from Kh-50-17-19, all in VBGI); (**I, J**) B. trichophyllum x hybrid1 (**I** from S-18-24-17, **J** from Kh-58-4-19); (**K, L**) B. neglecta (**K** from Prim-16-22-16, **L** from S-48-18-16, all in VBGI).

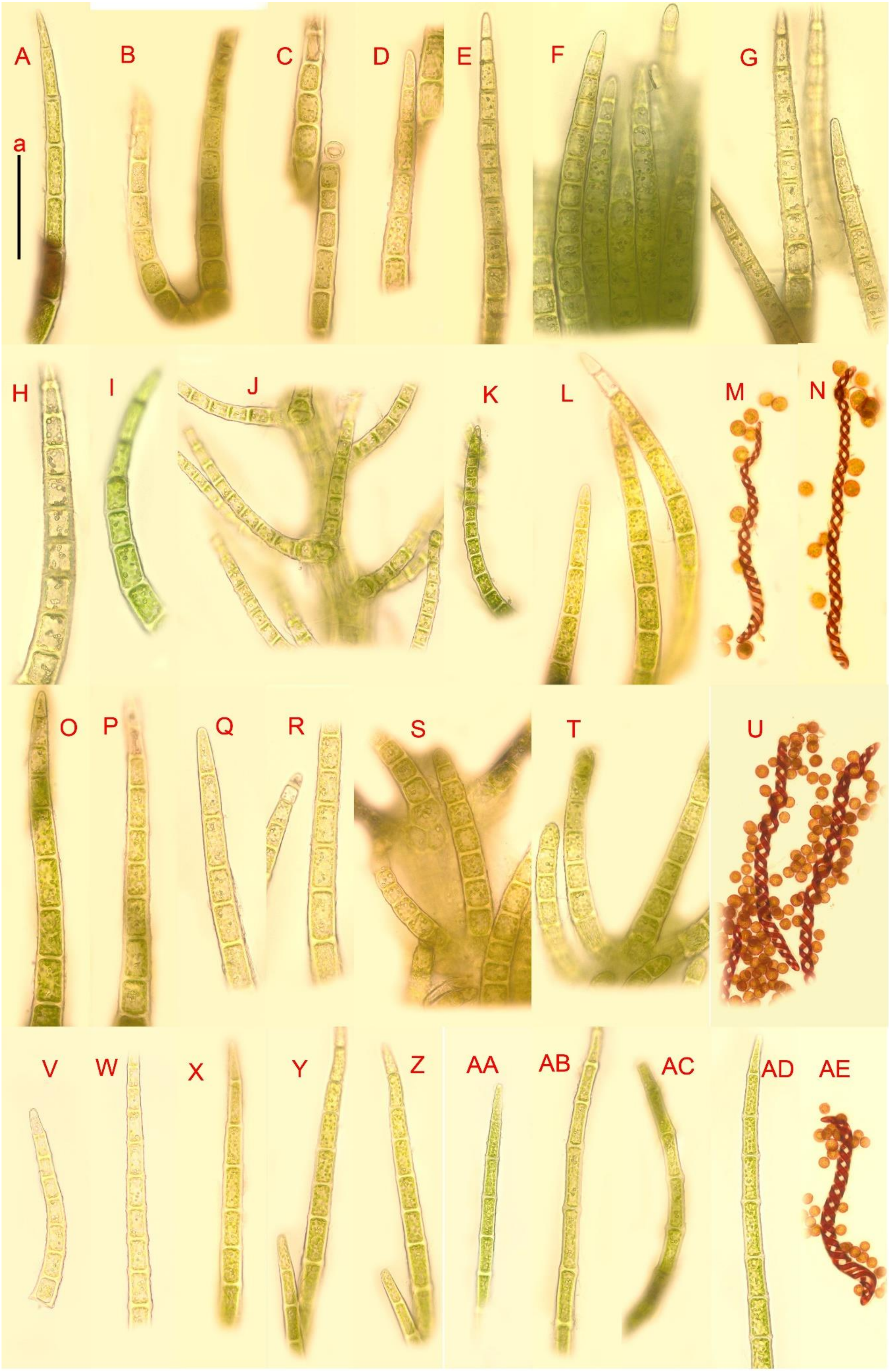
A
B
C
D
E
F
G
a
H
I
J
K
L
M
N
O
P
Q
R
S
T
U
V
W
X
Y
Z
AA
AB
AC
AD
AE

**Figure 2.** (**A–D**) *Blepharostoma prima* (**A** from S-45-12-16, **B, C** from Kh-47-28-19, **D** from S-14-17, all in VBGI); (**E–H**) *B. brevirete* (**E, F** from Mur-34-14-15, **G, H** – from K-50-11-15, all in VBGI); (**I**) *B. epixylica* (from J-11-13-15, VBGI); (**J, K**) *B. minor* (**J** from V-1-27-17, **K** from J-91-51-15, all in VBGI); (**L–R**) *B. trichophyllum* (**L–N** from Kh-46-12-19, **O, P** from Kh-50-17-19, **Q, R** from S-25-8-17, all in VBGI); (**S, T**) *B. trichophyllum x hybrid1* (from kh-58-4-19, VBGI); (**U–AE**) *B. neglecta* (**U, AE** from Kh-46-3-19, **V, W** from C-44-10-17, **X–Z** from Kh-46-3-19, **AA** from S-48-18-16, **AB, AC** from S-14-7-17, **AD** from Prim-16-22-16, all in VBGI). **A–L, O–T, V–AD** – leaf segments; **M, N, U, AE** – elaters and spores. Scale a – 100 μm.
